# Using Social Media to Uncover Treatment Experiences and Decisions in Patients With Acute Myeloid Leukemia or Myelodysplastic Syndrome Who Are Ineligible for Intensive Chemotherapy: Patient-Centric Qualitative Data Analysis

**DOI:** 10.2196/14285

**Published:** 2019-11-22

**Authors:** Alison Booth, Timothy Bell, Sonia Halhol, Shiyu Pan, Verna Welch, Evie Merinopoulou, Dimitra Lambrelli, Andrew Cox

**Affiliations:** 1 Evidera London United Kingdom; 2 Pfizer New York, NY United States

**Keywords:** social media, health-related quality of life, patient-centric, leukemia, myeloid, acute, myelodysplastic syndromes, natural language processing, patient preference, qualitative research

## Abstract

**Background:**

Until recently, treatment options were limited for patients with acute myeloid leukemia and myelodysplastic syndrome (AML and MDS) who are ineligible for intensive chemotherapy. Owing to the condition’s rapid progression, it is difficult to identify what is most important to patients when making treatment decisions. Patients’ needs can be better addressed by gaining a deeper understanding of their perspectives, which is valuable in the decision-making process. The Food and Drug Administration recently encouraged the use of social media as a tool to gain insight on patients’ perspectives regarding symptoms experienced and the impacts of their disease.

**Objective:**

This study aimed to use disease-specific social media posts by patients with AML or MDS who are ineligible for intensive chemotherapy and their caregivers to capture factors they feel are most important, and to provide current evidence to inform and characterize these perspectives.

**Methods:**

Posts by patients with AML or MDS and their caregivers were extracted from publicly available discussions on 3 large AML- or MDS–specific sites. These posts were manually reviewed to only include patients who are ineligible for intensive chemotherapy. A total of 1443 posts from 220 AML patients/caregivers and 2733 posts from 127 MDS patients/caregivers met the study inclusion criteria. A qualitative data analysis (QDA) of a sample of 85 patients’/caregivers’ posts was conducted to identify themes, and a targeted QDA of posts from 79 users focused on treatment decision discussions. Posts were manually reviewed, and relevant text segments were coded and grouped into categories and overall themes.

**Results:**

Eighty-six percent (73/85) of users in the overall QDA had relevant information about the key objectives. The most commonly discussed treatment experience theme was the humanistic burden of AML or MDS in terms of emotional/physical impact and impact on family (86%, 63/73 of users), followed by treatment decisions (56%, 41/73) and unmet needs (50%, 37/73). In the QDA of treatment decisions, 60 posts from 45 users contained relevant information. Patients commonly reported the desire to reach specific milestones, including birthdays and weddings. They wished for a better quality of life over quantity of life, did not want the risk of suffering from side effects, and expressed a clear preference to be at home rather than in a hospital or care home.

**Conclusions:**

This study was a novel application of disease-specific social media. It highlighted experiences in the current treatment of AML and MDS, including information gaps, patient/caregiver uncertainty, and the importance of understanding patients’/caregivers’ goals and opinions. A clear finding from this research was the importance of reaching certain personal life goals and being at home with family and friends. The analysis showed that patients/caregivers face additional challenges, including humanistic impacts and a lack of information regarding treatment options.

## Introduction

### Background

Myelodysplastic syndromes (MDSs) constitute a heterogenous group of hematopoietic stem cell disorders [1] in which the bone marrow produces immature white blood cells, red blood cells, platelets, or a combination of all three. MDS can be rapidly progressive and is associated with a risk of evolution into acute myeloid leukemia (AML) [2]. AML, an orphan disease, is an aggressive cancer of the blood, consisting of a group of relatively well-defined hematopoietic neoplasms [1]. The disease begins as abnormal blood cells that are produced in the bone marrow and can spread throughout the circulatory system and beyond if not diagnosed and treated quickly [3]. Both AML and MDS are hematologic diseases that generally affect older adults; they are uncommon before the ages of 45 and 50 years and are most commonly diagnosed in patients who are in their late 60s or 70s [4,5].

The annual incidence of AML in the United States is approximately 3.75 cases per 100,000 people, and the incidence rate of MDS is approximately 3.30 cases per 100,000 persons [1]. White men have the highest incidence of MDS (6.9 cases per 100,000 people) [6]. In 2018, there were an estimated 19,520 new cases of AML, and there are approximately 14,275 new cases of MDS annually according to data from 2010 to 2014 in the United States [6]. The number of new cases diagnosed each year is likely to increase as the size of the elderly population increases.

Prognosis is poor for patients with AML and MDS, and effective treatment options are limited. Prognosis for AML generally decreases rapidly with age—5-year survival in adults is estimated to be around 24%; however, this drops to less than 5% in individuals aged 65 years and older [7]. The treatment approach for MDS depends on several factors, including the type and risk group of MDS and the patients’ age and overall health. Most diagnosed patients are older or in poor health and, thus, are not suitable candidates for a stem cell transplant, which is potentially the only cure for MDS. Alternative treatments for MDS, such as supportive care (transfusions or blood cell growth factors) along with chemotherapy, serve only to relieve symptoms and avoid complications and side effects. The standard treatment for patients with AML is intensive chemotherapy, typically involving cytarabine-based regimens, with the goal being to achieve complete remission. Intensive chemotherapy commonly requires prolonged hospitalization as it is associated with severe myelosuppression and an increased risk of treatment-related mortality. As a consequence, elderly patients with AML who present with poor performance status and/or comorbidities are generally not considered candidates for intensive chemotherapy because the risks are thought to outweigh the benefits. Until recently, treatment options for these patients were limited to clinical trials, nonintensive agents (hypomethylating agents or low-dose cytarabine), or best supportive care [8]. Reported treatment patterns in older AML patients vary; however, estimates using data from 2000 to 2014 suggest that only 40% to 60% of these patients receive any therapy for their disease [9], in part, because of a lack of options, but other reasons are not well understood [10,11].

Owing to poor prognosis and limited treatment options for patients with AML and MDS ineligible for intensive chemotherapy, there are corresponding unmet medical needs. These conditions also impair patients’ health-related quality of life (HRQoL) and are associated with high symptom burden [12]. Patients’ needs can be better addressed by gaining a deeper understanding of their perspectives on the disease, treatment options, and the impact of both on their lives and those of their caregivers. This information is valuable in the decision-making process—including end-of-life treatment decisions—but is challenging to capture using traditional methods or data sources (eg, electronic medical records and patient questionnaires) because of the rapid progression of the disease and patients’ age at diagnosis. Recent studies have highlighted the need to evaluate patient-reported outcomes for symptoms of AML or MDS to outline areas of focus for clinicians and researchers [13-15]. However, studies assessing experiences, preferences, and perspectives regarding treatment decisions for patients with AML or MDS are scarce, especially for those who are ineligible for intensive chemotherapy [16,17]. Those studies are crucial to better inform drug discovery and regulatory decisions and imperative for improving the patient experience.

Disease-specific platforms (eg, patient forums and discussion boards) on social media represent a virtual community where patients and their caregivers spontaneously share experiences and perspectives related to their disease and treatments. This information can provide an instant snapshot of the current humanistic and economic burden of illness in patients with AML or MDS and provide insight into the rationale for patients’ treatment decisions. The Food and Drug Administration reinforced this idea in June 2018, when it made recommendations encouraging the use of social media to shed light on the patient’s perspectives regarding symptoms and impacts of their diseases, stressing the opportunity to inform medical product development and enhance regulatory decision making [18].

### Objectives

The objective of this study was to use social media on disease-specific platforms to identify themes related to disease and treatment experiences of patients with AML or MDS who are ineligible for intensive chemotherapy and their caregivers. It aimed to capture the most important factors that drive patients’ treatment decisions by identifying patients’ priorities and their reasons for pursuing certain types of treatments over others, including preferences in end-of-life situations.

## Methods

### Data Source

An initial search of disease-specific social media was conducted, focusing on the feasibility of addressing the study objectives (finalized on April 23, 2018). The search strategy focused on identifying AML and MDS–specific social media forums and discussion boards, using key search terms such as *AML patient forum* and *MDS patient discussion*. An exhaustive list of search terms used can be found in Multimedia Appendix 1.

Generic social media sites such as Facebook, Google+, and Twitter were not considered because of the added complication of filtering out irrelevant material and because access to data is governed by complex terms and conditions and data access fees. Searches were conducted using US and UK Google search engines, the first 5 pages of results were screened by title, and relevant forums were summarized. The searches yielded 41 sites for consideration for use in the study, which were assessed based on the following criteria:

- Each user has a username within the site, and the user can be easily classified as either a patient with AML or MDS or their caregiver/family member. The site topic must be clear, and posts must be specific to AML or MDS.
- The information contained in the forum must be relevant to the key study objectives (ie, disease symptoms and impacts on patients’ HRQoL, treatment sequence, treatment patterns, adverse events [AEs], and impact on caregivers).
- The forum site must be active and contain posts from recent years.
- The information must be posted in English.
- Material is freely available for anyone to find and read, and no registration is required.
- Posted content must be programmatically retrievable, without any restrictions from the site’s data protection terms and conditions.

Of the 41 sites identified during the initial search, 19 met the selection criteria. The following 3 forum sites with the highest number of posts were selected for this study:

1. AML Online Support Group: an active discussion group for patients with AML—visitors to the site are mainly from India, the United States, Pakistan, Indonesia, and Canada [19,20].
2. MacMillan Cancer Support: AML Online Forum: a support and discussion group for AML patients—visitors to the site are predominantly located in the United Kingdom but are also in the United States, India, Canada, and Australia [21].
3. MDS Foundation: MDS Patient Message Board: a platform where patients are encouraged to ask questions and get answers from fellow patients or MDS experts—the majority of its visitors are from the United States [22].

### Social Media Content Extraction and Data Preparation

Data extraction algorithms specific to each site were written in the R computational programming language (version 3.5.1 [23]) to extract posts programmatically from each site. Data extracted included username, post content, thread title, date of the post, and URL. A total of 44,456 posts (AML=21,654 and MDS=22,802) were extracted from 7811 users (AML=6373 and MDS=1438).

All data were programmatically deidentified at the time of extraction, and postal codes and addresses, email addresses, Web links, and telephone numbers were all removed. Following extraction, the data were processed and cleaned. Duplicate posts and non-Unicode Transformation Format-8 characters were removed. Misspellings were corrected using a dictionary of known misspellings in disease-related social media data compiled by the authors. The R hunspell package [24] was then used to identify additional misspellings. Despite these efforts, it is possible that some misspellings remained in the final data. Data were then assembled into individual user posting history files in .txt file format. These files contained all post content from each user chronologically. Extracted posts with the username *deleted_user* could not be assigned to user posting histories. Therefore, these were retained as individual posts and assumed to be individual users.

### Study Population

This study focused on patients with AML or MDS who were ineligible for intensive chemotherapy and their caregivers. The inclusion and exclusion criteria are provided in Textboxes 1 and 2, respectively. Intensive and nonintensive chemotherapies are listed in Textbox 3.

**Inclusion criteria.**

- Patients/caregivers of patients with acute myeloid leukemia/myelodysplastic syndrome who mention that they receive a nonintensive chemotherapy
- Patients/caregivers of patients with acute myeloid leukemia/myelodysplastic syndrome who mention that they are not treated with intensive chemotherapy
- Patients/caregivers of patients with acute myeloid leukemia/myelodysplastic syndrome who mention that they are treated with an intensive chemotherapy at a low dose
- Patients/caregivers of patients with acute myeloid leukemia/myelodysplastic syndrome who mention that they are ineligible for intensive chemotherapy

**Exclusion criteria.**

- Patients/caregivers of patients with acute myeloid leukemia/myelodysplastic syndrome who mention that they are treated with an intensive chemotherapy and who do not mention dose
- Patients/caregivers of patients with acute myeloid leukemia/myelodysplastic syndrome who mention that they have been treated with an intensive chemotherapy and who do not mention that they are no longer eligible to receive it
- Patients/caregivers of patients with acute myeloid leukemia/myelodysplastic syndrome for whom treatment received is not clear

**Chemotherapy treatments used to treat acute myeloid leukemia and myelodysplastic syndrome by intensity used in consideration of inclusion/exclusion criteria. Brand names are listed in parentheses for each generic drug.**

Intensive chemotherapy treatments

- Amsacrine (Amsidine)
- Arsenic trioxide (Trisenox)
- Cytarabine (cytosine arabinoside and high-dose cytarabine)
- Daunorubicin (Cerubidin)
- Etoposide (Eposin, Etopophos, and VePesid)
- Fludarabine (Fludara)
- Gemtuzumab ozogamicin (Mylotarg) + intensive chemotherapy
- Idarubicin (Zavedos)
- Mitoxantrone
- Tioguanine (thioguanine and 6-tioguanine)
- Tretinoin (Vesanoid and all-trans retinoic acid)

Nonintensive chemotherapy treatments

- Azacitidine (Vidaza)
- Decitabine (Dacogen)
- Gemtuzumab ozogamicin (Mylotarg)
- Low-dose cytarabine

#### Sample Size and Saturation of Emerging Themes

Saturation (the point at which no new significant themes emerge from the analysis) was judged and assessed by the research team. At various points throughout the analysis, the team discussed and decided to either add more samples (patient posting histories) from the generated pool of 347 study-eligible patients or stop those analyses if it was felt that saturation had been reached. This was independently done on an objective-by-objective basis.

#### User Classification

From the user posting histories compiled, an initial sample of posts from approximately 11.42% (892/7811) of randomly selected users was manually reviewed to determine whether the user met the inclusion criteria. The decision to include or exclude a user was based on phrases in post content (ie, “I have AML and am not eligible for intensive chemo”), and discussion of receiving one of the therapies is listed in Textbox 3.

#### User Selection

For the identification of themes related to disease and treatment experiences of patients, the research team judged that saturation had been reached and stopped further analysis at 80 AML user posting histories and 5 MDS user posting histories. Search terms relevant to end-of-life decisions were generated (details can be found in Table 1) to capture the most important factors that drive treatment decisions for patients, focusing on those that would help address the study objectives; posts containing any 1 of these search terms were included. For the research objective on treatment decisions, it was felt that saturation had not been reached. Therefore, posts of users included following the manual classification were further searched to maximize the relevant content available for analysis, and only those that contained specific search terms listed in Table 1 were included (79 users).

**Table 1:** Search terms used in targeted qualitative analysis.

Category	Search terms
End-of-life	*end of life, palliative, hospice, comfort care, and passed away*
Treatment	*no more treatment, stop treatment, not having any treatment, stopped treatment, no longer having treatment, and withdrawn*
Chemotherapy	*no more chemo, stop chemo, not having any chemo, stopped chemo, and no longer having chemo*
Living longer	*quality of life, keep living, live longer, stop living, determined, worth living, keep fighting, living for, more options, affairs sorted, come home, came home, be at home, and now at home*

### Analysis

Following the manual review of poster history files, qualitative data analysis (QDA) was undertaken for each of the objectives. Posts were qualitatively reviewed in the R Qualitative Data Analysis Package. Thematic analysis was used, taking a top-down approach to data, using hypothesis coding (themes and codes determined a priori) to develop a coding dictionary based on the key objectives [25]. Text segments relevant to the objectives were highlighted, and open coding was used to add codes based on reviewed data. The occurrence of themes was summarized using descriptive statistics.

## Results

### Study Population

**Figure 1:**
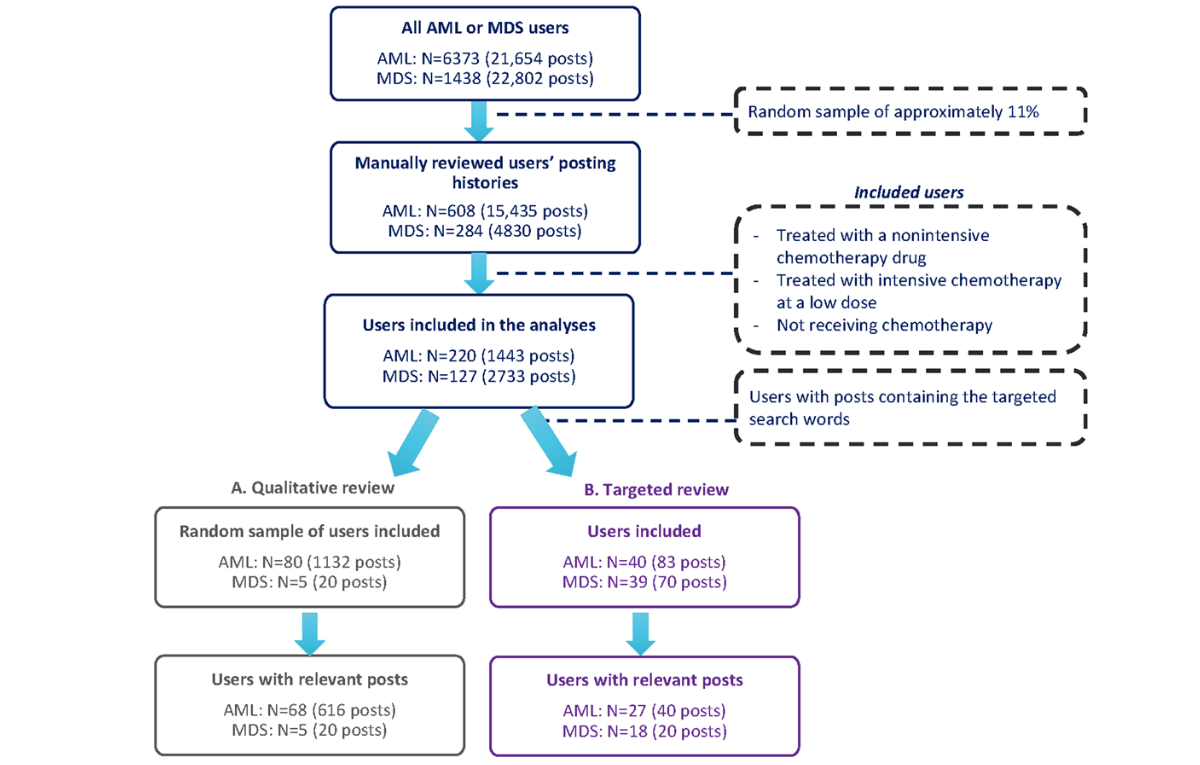
Study flow. AML: acute myeloid leukemia; MDS: myelodysplastic syndrome.

The posting histories of 892 users (AML=608 and MDS=284) including 20,265 posts (AML=15,435 and MDS=4830) were manually reviewed against the inclusion and exclusion criteria of the study, and, ultimately, 347 users (AML=220 and MDS=127) and 4176 posts (AML=1443 and MDS=2733) were included in the study. For the qualitative review, the 85 users included in the study contributed 1152 posts (AML=1132 and MDS=20). Of the 85 users, 73 (AML=68 and MDS=5) had posts relevant to the study. For the targeted qualitative analysis, 79 users (AML=40 and MDS=39) had posts containing the search terms, and on manual review of posts, 45 users (AML=27 and MDS=18) had posts relevant to the study objectives. For further details, see Figure 1.

### Identified Themes Related to Disease and Treatment Experiences

The following 5 overarching themes were identified from the results of the QDA: humanistic burden, treatment decisions, unmet needs, life milestones, and economic burden. The number and proportion of users mentioning each theme are shown in Table 2.

**Table 2:** Themes mentioned by the largest proportion of users.

High-level themes/subthemes		Description	Users discussing themes, n (%)
**Overall QDA^a^ (N=73)**			
	Humanistic burden	Impact on social life, impact on family, inability to do day-to-day activities, physical impact, and psychological impact	63 (86)
	Treatment decision^b^	Reasons for declining or not having treatment, reasons for pursuing one treatment over another, and stakeholders involved in the treatment decision	41 (56)
	Unmet needs	Emotional support, management of symptoms and side effects, and treatment options	37 (50)
	Life milestones	Spending time at home during the end-of-life period, putting affairs in order, and family and social events	14 (19)
	Economic burden	Medical expenses, travel expenses, and inability to work	9 (12)
**Treatment decision–targeted QDA (N=45)**			
	Health-related quality of life	Severity of side effects, ability to leave hospital, ability to travel, and being able to socialize	20 (44)
	Home and family	Being at home, reaching specific events, and spending time with family	19 (42)
	Physician decision	What the physician thinks is the best treatment option for the patient	5 (11)
	Patient and family wishes	What the patient and their family want to do regarding treatment	4 (9)
	Affairs in order	Finances, childcare, and writing wills	4 (9)

^a^QDA: qualitative data analysis.

^b^A total of 16 users included in the overall QDA had posts in the targeted treatment decision search; therefore, the total number of users mentioning the treatment decision theme in the study was 70. However, as no targeted search was conducted for the other themes, this number is not compared in the table above.

#### Humanistic Burden

The humanistic burden of AML and MDS was felt strongly by patients and caregivers and sometimes affected daily activities and HRQoL. Patients with AML or MDS were mostly burdened by physical impacts of the disease and/or treatment and most frequently mentioned feeling fatigue or weakness, followed by experiencing infections and fever. Patients experienced AEs that included coughing, headaches, loss of appetite (and subsequent weight loss), nausea and vomiting, pain, mouth blisters, nose bleeds, and hair loss. Many of these impacted daily activities such as eating, sleeping, and going out:

My mother is very frail and suffers with fatigue. She also has an infection so her doctors are holding off on any chemotherapy.

Caregiver

Posts by patients with AML or MDS revealed various psychological and emotional impacts of the disease. On diagnosis, many patients felt scared and anxious about their upcoming treatment. Many patients and their caregivers were unfamiliar with the disease before diagnosis, and they were left feeling confused because of their lack of knowledge about the disease and treatment options; in turn, this made them more likely to feel desperate and hopeless. The disease and/or treatment sometimes took a toll on patients’ cognitive abilities, affecting their concentration and memory as well as their mood:

I am very anxious at the moment and am not sleeping because I am waiting for my test results. I’m scared that I may not have much longer left.

Patient

Every day I feel fatigued, cannot concentrate and generally feel down. I’ve also begun to struggle with anger.

Patient

When my treatment stopped I was on the verge of having a breakdown, and since then have also been diagnosed with depression and PTSD. I also struggle sleeping and with nightmares so I am taking anti-depressants.

Patient

Family members felt an increasing responsibility to share caring duties and to provide emotional and financial support for patients with AML or MDS. The emotional impact on family was described as heartache, feelings of stress, confusion, fear, and guilt. As caregivers, patients’ families sometimes had to assume additional responsibilities such as making decisions on behalf of the patient, looking after the home, handling paperwork, and taking care of the patient and other relatives. This additional pressure on caregivers sometimes impacted their emotional and psychological well-being as well:

I have felt desperate and helpless, and I find it difficult to talk to friends about. It’s difficult to manage visits to the hospital as well at the same time as working.

Caregiver

I feel that I am useless to my husband, who now has to care for the children, me and manage our finances. I worry that he will resent me.

Patient

#### Treatment Decisions

The results of the QDA further categorized treatment decisions into the following 5 subthemes: HRQoL, home and family, physician decision, patient and family wishes, and putting affairs in order. The number and proportion of users mentioning each theme are provided in Table 2. The themes mentioned by the largest proportion of users were HRQoL and home and family (20/45, 44% and 19/45, 42%, respectively). The themes mentioned less commonly were patient and family wishes and putting affairs in order (both were 4/45, 9%).

Patients who discussed reasons for declining or not receiving chemotherapy and the stakeholders involved in the decision commonly mentioned avoiding potential AEs to maintain HRQoL as an important factor in their decisions. Patients (and their caregivers) expressed that they would rather have a better HRQoL over quantity of life and did not want to take the risk of suffering from side effects in the time they had left; as a result, some patients preferred not to receive treatment:

The doctors decided not to give him more chemotherapy the side-effects outweighed the benefits and threatened his life [...] the physician offered a chemotherapy with no side effects, but he does not want the risk. He is stronger and on good days we walk and go for drives, but he does get tired.

Caregiver

My mom said that the quality of her life is a priority over quantity. because of this she does not want to try the azacitidine[...] it makes me sad but her decision makes sense and I respect it.

Caregiver

In many cases, it was the physician who decided that a patient would not receive any treatment. Sometimes this aligned with the wishes of the patient and family; however, in some posts, it was clear that the patient/caregiver disagreed with the physician. They felt that the physicians did not want to fight the disease for the same amount of time as they did:

It felt like the doctors gave up before the patient did, so I kept fighting with my father and for my father.

Caregiver

For patients who discussed the reasons for pursuing certain types of treatments over others and the stakeholders involved in this decision, the most commonly reported reason for choosing a certain treatment was the desire for the patient to be at home rather than in a hospital or care home; this was expressed by both patients and caregivers. Treatments that allow patients to be at home and offer a comparable prognosis were the preferred option:

She has the option to go to a hospital closer to home and be able to go home every day. She said that all she wants is to be able to go home. This chemotherapy treatment will let her go home and give her as good prognosis.

Caregiver

As would be expected, physicians’ opinions played an important role in pursuing a particular treatment. Caregivers did not always agree with the treatment decision recommended by the physicians, and in some cases, felt that physicians were going too far and compromising the patients’ quality of life (QoL). The patient’s own wishes regarding their treatment—to explore all options and fight until the very end or to stop treatment—were also an important factor:

He had multiple rounds of azacitidine he isn’t improving [...] it seems like my father is their experiment, the doctors were offended when I said this. But his health and life quality has gone downhill and I am tired and annoyed and don’t know what to do.

Caregiver

My grandmother often felt weak, or was in pain, some days she cried or was confused, but, despite this, she did not succumb to it. Now, she’s on a ventilator in the hospital and I am fighting for her wish.

Caregiver

#### Unmet Needs

Patients and their caregivers expressed that they had run out of options or that there were not enough treatments available to them. The lack of treatments for elderly patients was also a topic of discussion, and some patients and caregivers expressed a view that age should not interfere with a patient’s ability to obtain treatment:

There aren’t a lot of solutions and no miracle drug.

Patient

Other posts identified the view that there was a lack of information and support from physicians. Some patients and caregivers expressed that they felt like their physicians did not provide adequate details needed for them to make informed decisions regarding treatments. Patients and caregivers also indicated a lack of empathy from physicians, which was perceived by them as the physicians refusing to continue treating the patients:

His results are being sent for a second opinion and so we can find a new doctor. our current oncologist isn’t helpful or supportive.

Caregiver

The doctor point-blank told her they wouldn’t resuscitate if something happened, almost like saying there was no point.

Caregiver

Patients and caregivers reported receiving inadequate information about specific treatments. Some mistakenly thought that the treatment would be a potential cure, and this sometimes led to disappointment and uncertainty regarding the effectiveness of the treatment and its impact on survival:

I was wrong thinking this was what would cure me, which was a stupid thought, rather than an opportunity to live.

Patient

No one is really sure how this treatment works, everything occurred so fast and wasn’t explained well, don’t know whether it’s because she doesn’t know how it works.

Caregiver

Overall, patients and caregivers expressed that there was a lack of information being provided to them regarding prognosis. They were often uncertain about what they had been told and needed clarity on the meaning of *poor prognosis*. Caregivers particularly mentioned a lack of warning about the potential for the disease to progress extremely rapidly and the way this could affect the patient. Some caregivers indicated that they would have made different decisions had they known this:

Doctors said he isn’t a candidate for high-dose chemotherapy so instead he’s getting a milder dose and he has a poor prognosis. what does this mean?

Caregiver

I’m trying to research disease progression because we’ve all been given broad ranges of days, weeks, or months.

Caregiver

When I went to see her at the hospital she didn’t recognize me. I didn’t expect this, nor did I think it would happen.

Caregiver

#### Life Milestones

Patient priorities and preferences in end-of-life situations revolved around being able to spend time with family/friends and was extremely important to both patients and caregivers. This included making the most of the time left or fulfilling the desire to spend their final moments with loved ones. Many patients wished to live long enough to reach a specific event (eg, wedding, Christmas, and birthday), and less frequently, they wanted to survive a specified amount of time that was not tied to a specific occasion:

It was his wish to live until Christmas and our wedding anniversary. he died in peace at home just when he wanted.

Caregiver

We have a vacation booked soon and we clearly want to go, it’s in another country. it’s the only opportunity we get to see our daughter a year.

Patient

Today was sad because there are no plans to bring my father home. i had hopes and plans of being together as a family for the weekend and things being normal.

Caregiver

Patients had to balance accomplishing certain tasks with the impact a treatment would have on their mental and physical capacity to conduct such tasks. Many patients prioritized putting their affairs in order in preparation for when they passed away, including finances (selling houses and settling bills), funeral plans, and arrangement for their family members:

I keep deteriorating. I am sorting out our assets, our land is being sold, I found new homes for our children and am taking care of my husband.

Patient

It is a strange situation: planning my funeral, writing my obituary, and taking care of other affairs, all while fighting with all of my power to live.

Patient

#### Economic Burden

The economic burden of AML or MDS was not frequently mentioned in the analyzed posts. When it was discussed, however, topics related to a lack of income because of the patient’s physical inability to work, caregivers not being able to work their standard hours because of additional pressures, and costs of treatment:

As a carer it’s very difficult, and sometimes I feel desperate, I have lost my job and now have to manage financial pressures too.

Caregiver

## Discussion

### Principal Findings

Patients’ perspective is a vital component to understanding treatment decisions patients make and better informing future decisions. The treatment landscape for patients with AML or MDS particularly lacks this frame of reference. Previous research has examined the definition of patients with AML or MDS who are ineligible for intensive chemotherapy, available treatment options, end-of-life care, and health care resource utilization [26-29]. However, no published research, to the best of our knowledge, has investigated patient preferences for treatment of this population or has explored the use of social media via disease-specific forums to research the experiences of these patients and their caregivers. This study was a novel design that combined the use of unstructured social media text and natural language processing with an established text analysis technique, QDA, to attempt to better characterize and understand the perceptions and priorities of patients with AML or MDS who are not eligible for intensive chemotherapy.

Although the focus of this study was patients with AML who are not eligible for intensive chemotherapy, some findings agreed with those of previously published studies that surveyed the more general AML population. The burden of physical impacts of the disease and/or treatment, including fatigue and weakness, infections, and fever, was frequently reported in this study and in published works that examined similar impacts [30,31]. This study also showed how important psychological and emotional impacts (eg, anxiety, depression, fear, and confusion) were to patients. These impacts were often caused by a lack of knowledge of the condition and its treatment or anticipated test results; these aspects were also reflected in previous studies [31].

Social media also provide caregivers and family members with a wider platform to discuss how they are impacted. This study demonstrated the impact on these individuals who were burdened by additional responsibilities to manage the patient’s affairs on top of their usual roles and duties. In turn, these burdens sometimes had a negative effect on the emotional and psychological well-being of the caregivers.

The study indicated that patients felt there was a lack of treatment options and did not feel that age alone should be a reason to not receive a particular treatment. There was also a clear theme around a general lack of information and knowledge regarding the condition and treatment options, resulting in the feeling that the patients and caregivers were unable to make fully informed decisions.

This study provided a unique perspective on the experiences and views of patients dealing with end-of-life situations, a situation not easily explored with a survey-based approach. Patients discussed goals that were broader and more complex than simply extending survival. Patients and caregivers highly valued time at home and with family or desired to reach certain life milestones or family events. These priorities might be considered less by clinicians and researchers, but their impact on mental well-being, survival, and HRQoL can be equally as important as qualitative markers and should be considered during the treatment decision-making process.

A recent survey of patients with AML concluded that treatment options that slow disease progression and do not compromise HRQoL are important in this population [15]. In keeping with this, our targeted analysis found that 44% of patients discussing treatment choices mentioned impact on HRQoL as a key influence in opting for one therapy over another. Surprisingly, aspects related to home and family (42%) were equally influential in choosing a treatment. Patients continued to emphasize the importance of being at home, reaching specific life events, and spending time with family. Interestingly, the negative effect of treatments on HRQoL was most cited in reasons for refusing certain treatments, with patients often stating a preference for higher HRQoL over quantity of life for this condition. Influences under the home and family theme were cited as the reasons for making or accepting certain treatment choices.

The use of social media offers a unique and complementary approach to capture the perspectives of patients and their caregivers. Published studies that aimed to characterize AML patients’ perspectives using a patient survey approach varied in sample size from 32 to 82 patients [30-32]. However, these studies looked at a range of AML patient groups and ages and covered signs, symptoms, impacts, positive and negative concepts, receiving information, and making treatment decisions. Buckley et al [30] and LeBlanc et al [32] recruited patients from a single hospital setting, which could have resulted in a lack of more general representation.

This social media study is the first, to our knowledge, to look specifically at the experience of AML and MDS patients ineligible for intensive chemotherapy, and the approach was uniquely adaptable to include more patients as needed. A pool of 347 patients qualified for this study—saturation was reached at 85 for the QDA of objectives other than treatment decisions, and 45 patients were used for a targeted analysis of treatment decision making. The sample sizes in this study were comparable with the largest of the published studies for AML and far exceeded guidelines for sample sizes discussed in the literature [31,33].

The use of social media has the added benefit of providing a more geographically diverse sample and reducing timelines for collecting information. Just as important, if not more so, this approach has the potential to collect unprompted responses by patients and caregivers. Many patients may be too weak or frail to participate in patient surveys and in health-related social media forums, whereas caregivers often participate on the patient’s behalf. The inclusion of the experiences of caregivers provides a unique and potentially more comprehensive perspective to the approach of using social media.

A recent publication compared the use of disease-related social media, concept elicitation interviews, and group concept mapping to understand the perspectives of patients with ankylosing spondylitis. The study found that disease-related forums on social media uncovered the most concepts/perceptions, used the least amount of resources, and allowed access to a larger amount of material [34]. Our study also found the use of social media to be an efficient and cost-effective way to collect data of this type. In addition, this study presented a novel insight into the experiences and opinions of a patient group that had not been previously studied. Considering the rarity of the condition and the difficulties in recruiting patients with AML who are not eligible for intensive chemotherapy, this study drew from a relatively large sample size for a more representative look at this patient group. Another strength of this study was that the themes and concepts were driven by the discussions between the patients and caregivers themselves—they were not guided by any moderator, medical professional, or researcher and are therefore less likely to be impacted by information bias than traditional questionnaire or interview studies. Instead, they offer a more accurate and novel reflection of issues that are most important to patients and caregivers.

Social media, however, are a fairly unregulated source of information and should be used with caution. Compared with traditional patient interview studies, the biases present in disease-related social media data are currently not well understood; therefore, it is difficult to characterize the types of bias that could impact the results of this study. However, it should be noted that no study is free of all bias.

### Conclusions

Social media provide a window into patients’ and caregivers’ perspectives using a previously untapped approach. The ability to get a more representative and current depiction of what matters to patients with AML or MDS and their caregivers can have an important influence on the treatment landscape and the resulting decision-making process.

This work highlighted the themes that are important to patients with AML or MDS and their caregivers, which have previously been given little consideration but, nonetheless, have a clear influence on their experiences and treatment decisions. The following themes were considered as very important: improved QoL over extension of life, being at home, spending time with family, and living to reach certain milestones. This study also confirmed what had been previously reported in the more general AML population—physical impacts on QoL such as AEs are important to patients and play a role in treatment decisions. Our approach, however, offered the unique ability to examine a wider range of unsolicited topics and provided a more in-depth look at these discussions.

Physicians and other medical staff should consider these *softer* preferences and goals and use this knowledge to engage in more comprehensive, informative discussions with patients and caregivers, especially given the lack of treatment options for this patient population. This knowledge also emphasizes the need to close the information gap for patients and caregivers regarding treatment options and can help clinicians make better-informed recommendations for their patients. Furthermore, the findings of this study can be used to inform further research in this population and allow for patients’ preferences to be taken into account during drug development and for regulatory decisions.

## Appendix

Multimedia Appendix 1 Search Terms Used to Identify acute myeloid leukemia (AML) or myelodysplastic syndrome (MDS) specific online forums.

## Abbreviations

AE: adverse event
AML: acute myeloid leukemia
HRQoL: health-related quality of life
MDS: myelodysplastic syndrome
QDA: qualitative data analysis
QoL: quality of life
