# Supplementary material for: Using Social Media to Uncover Treatment Experiences and Decisions in Patients With Acute Myeloid Leukemia or Myelodysplastic Syndrome Who Are Ineligible for Intensive Chemotherapy: Patient-Centric Qualitative Data Analysis
Source: J Med Internet Res. 2019 Nov 22;21(11):e14285. doi: 10.2196/14285 (PMC6898885; doi:10.2196/14285)
Supplement: Multimedia Appendix 1 [file jmir_v21i11e14285_app1.docx]

**Using Social Media to Uncover Treatment Experiences and Decisions in Patients with Acute Myeloid Leukemia or Myelodysplastic Syndrome who are Ineligible for Intensive Chemotherapy: A Patient-Centric Approach**

**Authors**

Booth A, MSc^1^, Bell T, MHA^2^, Halhol S, MSc^1^, Pan S, MSc^1^, Welch V, PhD, MPH^2^, Merinopoulou E, MSc^1^, Lambrelli D, PhD^1^, Cox A, PhD^1^

**Affiliations**

^1^Evidera, London, UK; ^2^Pfizer, New York, US

## Appendix 1

For the initial disease-specific social media search, the following terms were searched in Google UK and Google US search engines between April 16, 2018 and April 23, 2018:

- “acute myeloid leukaemia forum”
- “acute myeloid leukaemia patient forum”
- “acute myeloid leukaemia patient discussion”
- “aml forum”
- “aml patient forum”
- “aml patient discussion”
- “myelodysplastic syndrome forum”
- “myelodysplastic syndrome patient forum”
- “myelodysplastic syndrome patient discussion”
- “mds forum”
- “mds patient forum”
- “mds patient discussion”
